# Assessing Knowledge and Attitude of Dental Patients regarding the Use of Dental Implants: A Survey-Based Research

**DOI:** 10.1155/2019/5792072

**Published:** 2019-07-28

**Authors:** Abdulrahman Alajlan, Aryaf Alhoumaidan, Abeer Ettesh, Mazen Doumani

**Affiliations:** ^1^Dentist, College of Dentistry, Qassim University, Buraydah, Qassim, Saudi Arabia; ^2^Lecturer, Department of Oral & Maxillofacial Surgery & Diagnostic Sciences, College of Dentistry, Qassim University, Buraydah, Qassim, Saudi Arabia; ^3^Alfarabi Colleges of Dentistry and Nursing, Department of Restorative Dental Sciences, Riyadh, Saudi Arabia

## Abstract

**Aim:**

The purpose of this study was to assess the level of knowledge, attitude, and source of information regarding the use of dental implants as treatment option compared to other conventional treatment modalities.

**Materials and Methods:**

A descriptive cross sectional study among adult dental patients attended dental clinics of College of Dentistry, Qassim University. The level of knowledge, source of information, and attitude regarding the use of dental implants were evaluated through standardized self-explanatory questionnaires which were handed to the patient during their regular dental visits. 200 patients were selected randomly to be included in this survey.

**Results:**

Among the 200 participants included in this study, 91.5% of the respondents heard about implants and their source of information were friends (45.5%), 38% of the respondents had no idea about the oral hygiene for the care of the implants compared with natural teeth, 28.5% of the respondents expected them to last between 10 and 20 years, and 48.5% of the respondents believed that dental implants have no effects on the systemic health and there was nonsignificant difference between males and females.

**Conclusion:**

The survey concluded that an acceptable level of awareness regarding using dental implants as a treatment option for replacing missing teeth, with friends being the main source of information.

## 1. Introduction

Long-term clinical studies of dental implants have proved the effectiveness of implant treatment as an option to replace missing teeth [1]. A dental implant is an artificial root inserted surgically to support the complete denture or to replace single or maxillofacial prosthesis [2]. It is the best treatment option to replace single or multiple missing teeth [3]. It was originally used for the treatment of edentulous patients to improve denture retention, stability, and functional efficiency. Recently, it is widely accepted as a prosthetic treatment option compare to conventional methods [1]. The main reasons for choosing implant treatment were restoring lost teeth (35.5%), followed by the dentist's advice (33.3%) reported by Annibali et al. [4]. The rehabilitation including implants improves satisfaction of oral function [5]. Grogono et al. measured the psychological attitudes of patients to implant prostheses and compared their status before and after therapy, and they found that satisfaction with the implant prosthesis was significantly greater than the conventional treatment modalities. 88% of the respondents indicated that their confidence was improved [6]. Several studies have been conducted to show the patients' awareness about dental implants. A survey by Zimmer et al. [7] found a high awareness rate as well as general positive attitude toward oral implants therapy. In a study conducted in Saudi Arabia, Al-Johany et al. reported that 66.4% of patients were aware about dental implants, and the relatives and friends were the main source of information about dental implants for 31.5% of patients [8]. In another study conducted in Iran by Faramarzi et al., 60% of the subjects knew about dental implants and dentists were the main source of information about dental implants (42%) [9]. Awooda et al. found that 68.5% were aware about dental implants, and the main source of information about implants were relatives and friends (38.2%) [10]. So, the present study aimed at evaluating the level of knowledge, source of information, and attitude regarding the use of dental implants as a treatment option compared with other conventional treatment modalities. The purpose of this survey was to evaluate the level of knowledge, source of information, and attitude regarding the use of dental implants as a treatment option compared with other conventional treatment modalities.

## 2. Materials and Methods

A self-explanatory questionnaire was designed to assess the level of knowledge, source of information, and attitude of dental patients regarding using dental implants for replacing missing teeth which were in correspondence to previous studies conducted by Kohli et al., and the questionnaire comprises 16 questions to evaluate the level of knowledge of dental patients toward implant treatment, evaluate the source of information regarding dental implant treatment, and evaluate the attitude of dental patient toward using dental implants as a treatment option compared to other conventional treatment modalities. The questionnaires were distributed in dental clinics of Collage of Dentistry, Qassim University, Saudi Arabia. The questionnaires were handed to the patient during their regular dental visits. All the respondents were informed about the aim of the study. A random sampling method were carried out with convenient sample size (*n* = 200).

### 2.1. Study Design, Area, and Population

A descriptive cross sectional study was conducted among adult dental patients attended dental clinics of College of Dentistry, Qassim University. Data were collected between February 24, 2017, and March 15, 2017. Inclusion criteria were as follows: adults 20 years or more, not inpatient, and with no previous dental implants. Exclusion criteria were as follows: very old uncooperative patients, patients less than 20 years of age, and mentally or physically disabled patients.

### 2.2. Sampling Techniques and Size

A total of 200 (female: 100; male: 100) participants who fulfilled the required criteria during study period were studied. They were selected by the simple random convenience sampling technique. The questionnaires were handed to the patients during their regular dental visits. All the respondents were informed about the aim of the study.

### 2.3. Survey Tool

A self-explanatory closed-ended questionnaire was administered with a total of 16 items in three sections designed to assess the patient's knowledge, source of information, and attitude about using dental implants as a treatment modality for replacement of missing teeth, which were in correspondence to previous studies conducted by Kohli et al. [11] and Faramarizi et al. [9].

Demographic data, socioeconomic status, and level of education were assessed. The questionnaire was prepared bilingually (English and Arabic) to correspond with the reading and comprehension levels of patients with different levels of education. Eligible illiterate patients were interviewed. It took 7–10 minutes to answer all the questions, and the questionnaire was filled in the waiting hall of the dental clinic of Qassim University. A pilot study was conducted among a sample of 25 patients (fifteen literate patients by self-administration of the questionnaire and ten illiterate by interview) by using the structured questionnaire to ensure comprehensibility and reliability. These 25 questionnaires were not included in the final study.

### 2.4. Statistical Analysis

The collected data were cleaned, coded, entered in Excel, and analysed by using Statistical Package for Social Sciences (SPSS; IBM SPSS Inc., Chicago, version 15). The chi-square test was used to compare two categorical data in contingency table. Frequency tables were used to determine the proportion level of variables among surveyed patients, with the level of significance set at *P*=0.05.

### 2.5. Ethical Consideration

The study was approved by Ethical Committee in College of Dentistry, Qassim University (code: EA/201/2017). Selected patients were requested to participate voluntarily after explanation of the purposes of the study. Informed written consent for their participation was obtained and confidentiality of responses was assured. Those patients who had not heard of dental implants as a treatment option were educated in this regard.

## 3. Results

Two hundred patients were questioned during the study period. Among the 200 subjects, 50% were females and 50% were males.

### 3.1. Level of Knowledge and Attitude

91.5% (183) of the respondents heard about implants, 46% (92) were female and 45.5% (91) were male, while 8.5% (17) of subjects did not hear about implants before. There is significant difference between males and females (*P*=0.800) (Table 1).

Regarding the oral hygiene for the care of the implants compared with natural teeth, 38% (76) of the respondents do not have any idea (“no idea”) (48% were male and 28% were female), while 34.5% (69) thought that the implants need more care compared with natural teeth, 14% (28) thought both are similar, and 13.5% (27) thought that it needs less care compared with natural teeth. There was significant difference between males and females (*P* ≤ 0.001) (Table 2).

Regarding the durability of implants, 28.5% (57) of the respondents expected them to last between 10 and 20 years, 15.5% (31) of the respondents expected the durability between 21 and 25 years, and 15% (30) of the respondents estimated the durability to be less than 10 years. 12% (24) of the respondents estimated the durability to be more than 25 years, and 29% (58) of the respondents had no idea (Table 2).

In the most of the respondents, 45% thought that the functional outcome of dental implants was important, followed by 35% of the respondents thought that it is very important, while 2% of the respondents thought it is not very important and 26.5% of the respondents had no idea (Table 2).

Table 2 shows that 70% (140) of the respondents had experiences by themselves or heard about experiences from relatives and the outcome of the implant therapy was successful in 82.6% (123) of the respondents who had positive experience. There was nonsignificant difference between males and females (*P*=0.335).

48.5% (97) of the respondents believed that dental implants have no effects on the systemic health and most of them were female 54% (54), while 40% (80) of the respondents believed that dental implants have no effects on the systemic health. There was nonsignificant difference between males and females (*P*=0.090) (Table 2).

Most of the respondents (37 (36.5%)) answered that the effect of implant treatment in comparison with common prosthesis is important, while 53 (26.5%) of respondents had no idea. There was nonsignificant difference (*P*=0.223) (Table 2).

### 3.2. Source of Information

**Figure 1:**
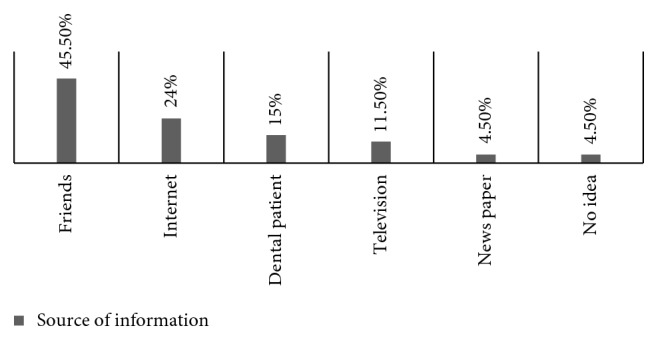
Percentage of different sources of information about dental implants as preferred by the questioned subjects.

Among the 200 respondents, the most common source of information was friends (91 (45.5%)), followed by dentists (72 (36%)), Internet (48 (24%)), dental patients (30 (15%)), and television (23 (11.5%)), and the newspaper was the least source of information (9 (4.5%)), and 9 (4.5%) had no idea (Figure 1).

## 4. Discussion

The present survey assessed the knowledge, source of information, and attitude of dental patients attending Qassim University's Dental Clinics regarding using dental implants as an option in replacing missing teeth. In the present study, most of respondents heard about implants (91.5%). Similar results were found by Alanazi et al. (90.6%) [12]. In contrast, Chowdhary et al. [13], Tomruk et al. [14], Kohli et al. [11], and Santhosh Kumar et al. [15] found that the many of them were unaware about using dental implants as an option for replacing missing teeth. In contrast, in the studies conducted by Suwal et al. [16], Awooda et al. [10], and Al-Johany et al. [8], 52.6%, 68.5%, and 66.4%, respectively, were aware of implant therapy.

This survey showed that the most common source of information was friends 91 (45.5%), and this is in agreement with the study conducted by Awooda et al. [10], Al-Johany et al. [8], and Suwal et al. [16], and they found that the main source of information about implants were relatives and friends (38.2%, 31.5%, and 30.2%, respectively). Many studies found that dentists were the main source of information of the subjects conducted by Esfahani and Moosaali [17], Kohli et al. [11], and Tomruk et al. [14] (40.7%, 53.6%, and 44.5% respectively). Zimmer et al. [7] found through a survey conducted in the USA that media and friends (77%) play a much more important role. Tapper et al. [18], Faramarzi et al. [9], and Alanazi et al. [12] reported that most of patients believed that using of implants needs more care (46%, 33%, and 66%, respectively), while the present study showed that 38% of the respondents do not have any idea, while 34.5% thought that the implants need more care compared with natural teeth. Regarding expected mean of durability of dental implants, Tapper et al. [18] showed 54% of patient believed expected mean durability of implant is 10–20 years. Esfahani and Moosaali [17] and Faramarzi et al. [9] reported that 37.7% and 70.7%, respectively, of the subjects had no idea about the durability of dental implant treatment, and also in the present study, 29% of the respondents had no idea about durability of implants while 37.7% of the subjects had no idea about the durability of dental implant treatment. This means patients had insufficient information about dental implants. Patient's expectations of improved function are the main reason for choosing an implant. Zimmer et al. [7] and Faramarzi et al. [9] reported that function is the most important factor. Similar results in this study, 45% of the respondents thought that the functional outcome of dental implants was important.

## 5. Conclusion

This study showed that most of dental patients who attended Qassim University Clinic aware about dental implants. It also showed the need for providing more general and correct information to the patients about dental implants. Friends was the main source of information. Further studies are needed with larger sample sizes to evaluate the level of information of the dental patients who attended governmental and private dental clinics in different areas in Qassim.

## Figures and Tables

**Table 1:** Respondents' knowledge about dental implants.

	Male, *n* (%)	Female, *n* (%)	Total, *n* (%)	Significance
Yes	92 (46)	91 (45.5)	183 (91.5)	*X* ^2^ = 0.064 *P*=0.800
No	8 (4)	9 (4.5)	17 (8.5)	

**Table 2:** Patient questionnaire to evaluate knowledge and expectations regarding implants.

	Male, *n* (%)	Female, *n* (%)	Total, *n* (%)	Significance
*What do you anticipate as oral hygiene for the care of implants compared with natural teeth?*				
Similar	13 (13)	15 (15)	28 (14)	*X* ^2^ = 22.076 *P* ≤ 0.001
More	20 (20)	49 (49)	69 (34.5)	
Less	19 (19)	8 (8)	27 (13.5)	
No idea	48 (48)	28 (28)	76 (38)	

*What do you estimate as the functional life of implants (years)?*				
<10	14 (14)	16 (16)	30 (15)	*X* ^2^ = 22.797 *P* ≤ 0.001
10–20	39 (39)	18 (18)	57 (28.5)	
21–25	18 (18)	13 (13)	31 (15.5)	
>25	3 (3)	21 (21)	24 (12)	
No idea	26 (26)	32 (32)	58 (29)	

*How important for you is the functional outcome of implant supported prosthesis?*				
Not very important	9 (9)	1 (1)	10 (5)	*X* ^2^ = 14.548 *P*=0.002
Important	52 (52)	38 (38)	90 (45)	
Very important	29 (29)	39 (39)	68 (34)	
No idea	10 (10)	22 (22)	32 (16)	

*Have you ever heard about experiences with implants from your friends?*				
Yes	67 (67)	73 (73)	140 (70)	*X* ^2^ = 0.857 *P*=0.335
No	33 (33)	27 (27)	60 (30)	

*If yes, how successful was the implant?*				
Successful	69 (93.2)	54 (72)	123 (82.6)	*X* ^2^ = 11.990 *P*=0.002
Partially successful	5 (6.8)	19 (25.3)	24 (16.1)	
Not successful	0 (0)	2 (2.7)	2 (1.3)	

*Have you ever heard about effects of dental implants on systemic health?*				
Yes	43 (43)	54 (54)	97 (48.5)	*X* ^2^ = 4.819 *P*=0.090
No	41 (41)	39 (39)	80 (40)	
No idea	16 (16)	7 (7)	23 (11.5)	

*How are the effects of implant treatments in comparison with common prosthesis treatments?*				
Not very important	2 (2)	2 (2)	4 (2)	*X* ^2^ = 4.381 *P*=0.223
Important	32 (32)	41 (41)	73 (36.5)	
Very important	42 (42)	28 (28)	70 (35)	
No idea	24 (24)	29 (29)	53 (26.5)	

## Data Availability

The data used to support the findings of this study are available from the corresponding author upon request.
